# Characterizing the complete mitogenome of *Odontothrips phaseoli* (Thysanoptera: Thripidae) and its mitochondrial phylogeny

**DOI:** 10.1080/23802359.2024.2386418

**Published:** 2024-07-31

**Authors:** Dongxue Wang, Chengwen Li, Lihong Dang

**Affiliations:** a School of Bioscience and Engineering, Shaanxi University of Technology, Hanzhong, China; b Shaanxi Province Key Laboratory of Bio-Resources, Hanzhong, China; c Qinba Mountain Area Collaborative Innovation Center of Bioresources Comprehensive Development, Hanzhong, China; d Qinba State Key Laboratory of Biological Resources and Ecological Environment (Incubation), Hanzhong, China

**Keywords:** Thrips, gene rearrangement, phylogenetic, Thripidae

## Abstract

Described originally from Heilongjiang, China, *Odontothrips phaseoli* is a potential pest of threatening bean plant in northern China. The complete mitochondrial genome of *O. phaseoli* was sequenced and assembled, with a total length of 15,540 bp. Within this genome, 37 genes have been identified: 13 PCGs, 22 tRNAs, two rRNAs, and two putative control regions. Most PCGs terminate with TAA, while four genes (*atp8*, *nad1*, *nad2* and *nad4*) use an incomplete ‘T’ and *nad6* employs TAG as the stop codon. Compared to the mitogenome of the ancestral insect, *O. phaseoli* displays significant gene rearrangement. However, it retains three conserved gene blocks in common with its related species, *Megalurothrips usitatus*, both of which belong to the *Megalurothrips* genus-group. The phylogenetic tree, constructed based on the entire mitogenome dataset of all thrips species available in NCBI, shows that the two species cluster closely together. This alignment might underscore the close link between gene arrangements and the phylogeny relationships.

## Introduction

The second most species-rich subfamily in Thysanoptera, Thripinae includes 1785 extant species in 231 genera (ThripsWiki 2023), of which many members are most of pest thrips and all of the tospovirus vectors. The species of flower-living genus *Odontothrips* are known as damaging alfalfa and vegetables of Fabaceae. Especially, *Odontothrips phaseoli*, described from Heilongjiang, China by Kurosawa (1941), is now considered as an important pest of bean plant of *Phaseolus* in northern China. In addition, in the eight genera of the *Megalurothrips* genus-group, *Megalurothrips* and *Odontothrips* are particularly closely related and confused (Mound and Palmer 1981; Xie et al. 2010). In morphology, the species of these genera share a pair of dorso-apical setae on the first antennal segment which is unusual in Thripinae (Hakimara and Minaei 2019), but it is difficult to distinguish them by morphology. Currently, the mitogenome of insect, as a molecular marker, is helpful to analyze the relationships of tribes and genera in many insects (Chen et al. 2019; Zhou et al. 2020; Chen et al. 2021; Wu et al. 2022). In Thysanoptera, some attempts were made to reveal phylogenetic and evolutionary relationships among genera and species with mitogenome data (Chakraborty et al. 2018; Kumar et al. 2019; Liu et al. 2022; Pakrashi et al. 2023). In NCBI, only one mitogenome of *Megalurothrips*, *M. usitatus*, is available but none of *Odontothrips* (Xing-Ming et al. 2023). To help understanding the relationship among them, the complete mitochondrial genome of *O. phaseoli* was sequenced, and the phylogenetic tree was constructed.

## Materials and methods

**Figure 1.**
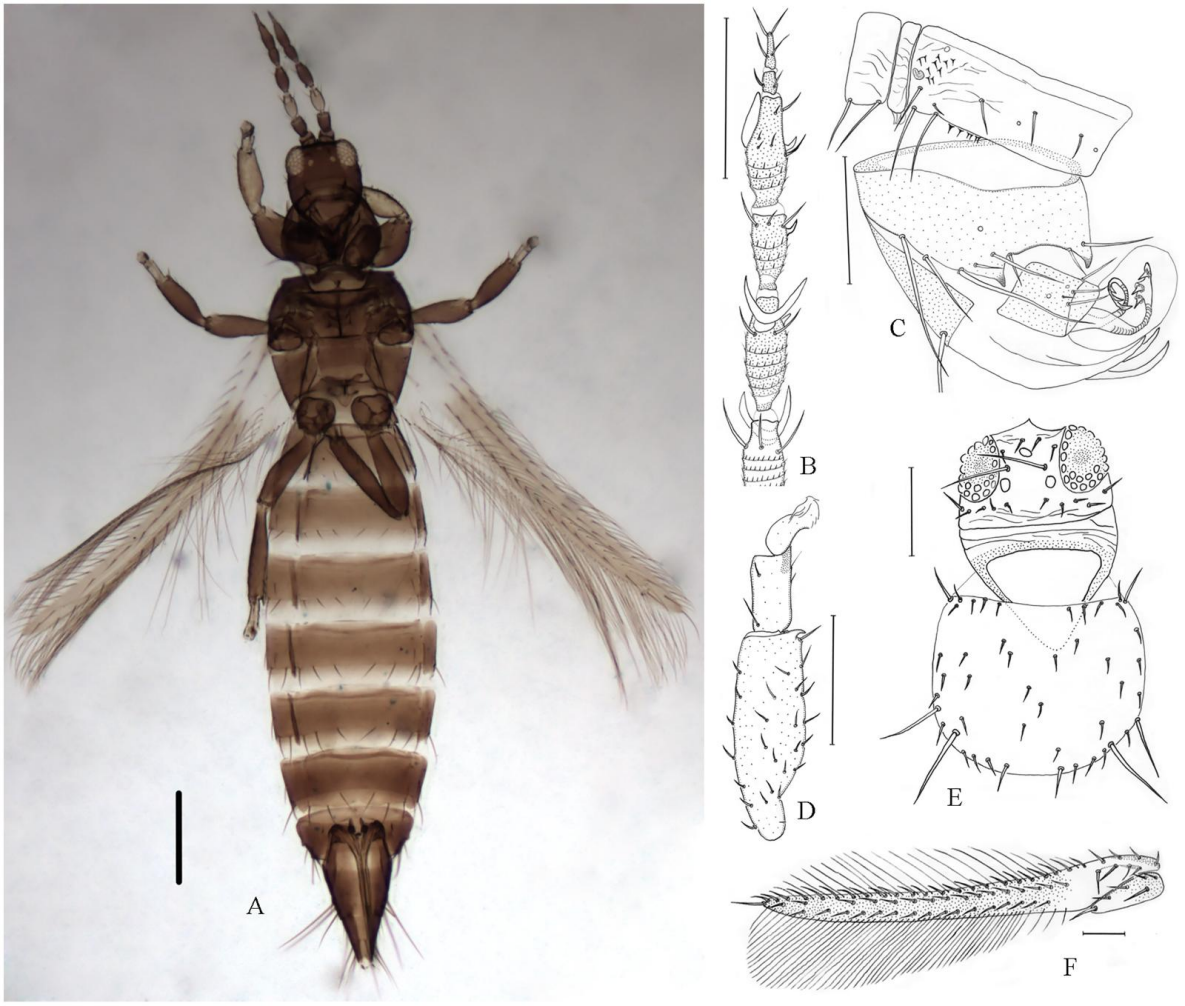
Dorsal view of *O. phaseoli*. (A) Body, female; (B) Antennal segments III–VIII; (C) Male tergite VIII–X with three endothecal spines at and near the apex of each canaliculus; (D) Fore tibiae and tarsal; (E) Head and pronotum; (F) Forewing. Scale bars = 100μm. Identified and photographed by Lihong Dang.

The samples of *O. phaseoli* were collected from flowers of *Phaseolus* in Shaanxi Province of China in 2021 (33°86′88″N, 109°94′74″E), and were preserved in 95% ethanol and stored at −20 °C for DNA extraction. The voucher specimens (No. JM2021030) were deposited at the School of Biological Science and Engineering, Shaanxi University of Technology (L.H. Dang, danglihong@snut.edu.cn). The diagnosis of *O. phaseoli* is as follows (Figure 1): ocellar setae S1 present, S2 much elongate; antennal segment VI with inner sense cone having a wide base at least one-third as long as the length of inner margin of this segment; pronotum with two pairs of major setae; fore tarsi without apical tubercle on inner surface; forewing brown with pale at base, first vein with 4 + 11–17 + 2 setae; fore tibiae with a large apical claw ventrally; antennal segment only III pale, other segments uniform brown; abdominal sternites without discal setae, IV with three pairs of posteromarginal setae arising at margin, VII setal pair S1 arise in front of margin, S2 and S3 arise at margin; male with 3 endothecal spines at and near apex of each canaliculus.

Total genomic DNA was extracted from a single thrips sample using the Biomarker Micro Cell/Tissue DNA Kit. And sent to BerryGenomics (Beijing, China) forsequencing, which was conducted using the Illumina NovaSeq 6000 platform (San Diego, CA, USA) with 150 bp paired-end reads. Finally, a total of 4.24 Gb clean data was used to assembly by NOVOPlasty (Dierckxsens et al. 2017) with default parameters. PCGs and RNAs of the complete mitogenome was firstly predicted using MITOS2 web server (Bernt et al. 2013), and then manually annotated and corrected using Geneious v. R9 software (Kearse et al. 2012). The AT-rich control regions were identified *via* boundaries of adjacent genes. Nucleotide composition, codon usage was estimated in PhyloSuite v1.2.2 (Zhang et al. 2020). Circular map of the mitogenome was drawn with CGView Server (Grant and Stothard 2008).

The whole mitogenome dataset (13PCG + 22tRNA + 2rRNA) of 34 thrips species including the target species were aligned by MAFFT v7.313 (Katoh and Standley 2013) in PhyloSuite v1.2.2 (Zhang et al. 2020), with *Aphis gossypii* (Zhang et al. 2016) and *Alloeorhynchus bakeri* (Li et al. 2012) as outgroups. MACSE (Ranwez et al. 2018) was utilized to optimize the alignments of protein-coding genes, while the protein-coding genes and RNA genes were trimmed by Gblocks (Talavera and Castresana 2007). BI (Bayesian Inference) tree was reconstructed by MrBayes (Ronquist et al. 2012). The resultant tree was visualized in iTOL (https://itol.embl.de/).

## Results

**Figure 2.**
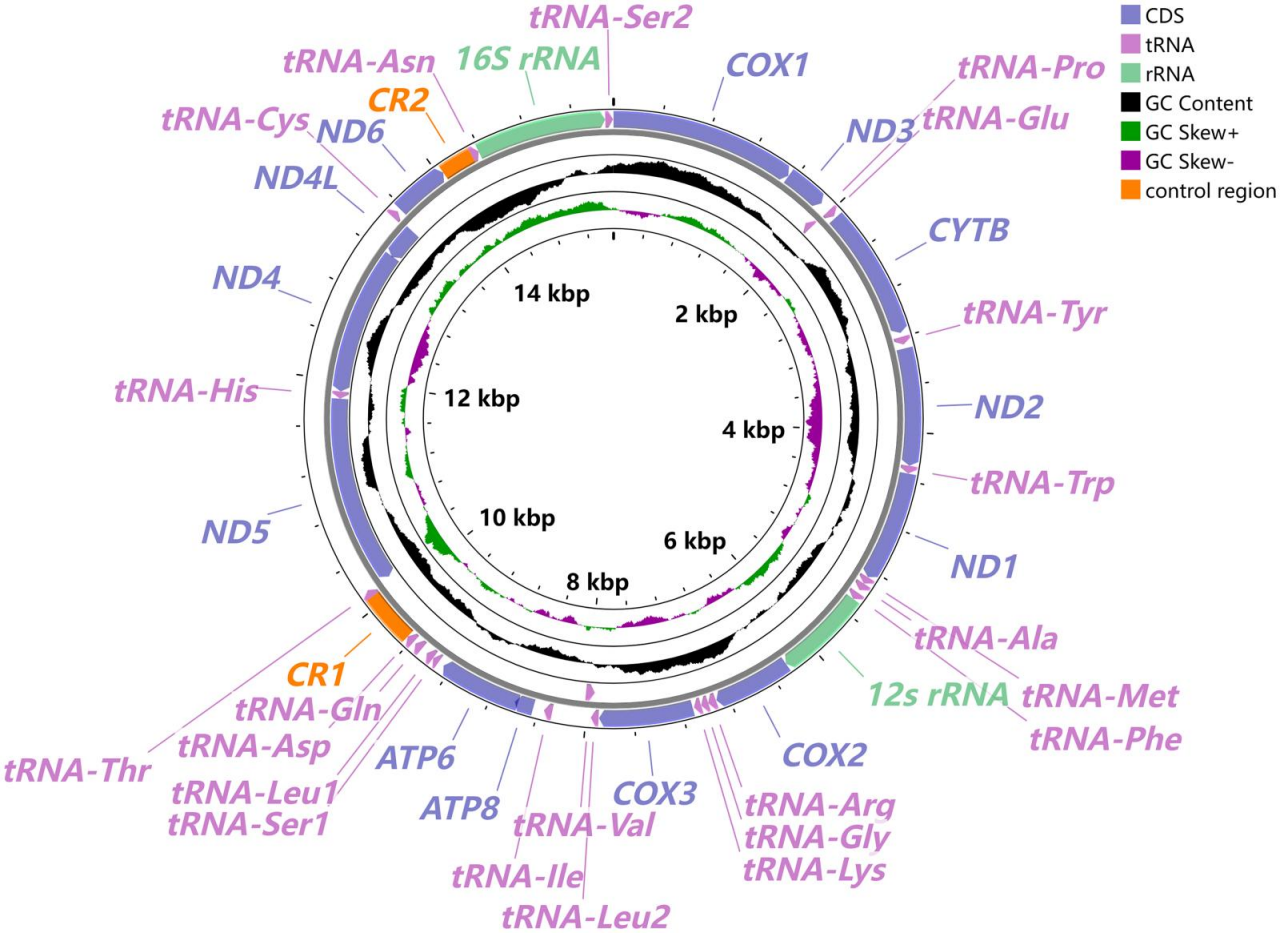
The circular representation of the complete mitogenome of *O. phaseoli*. The innermost and Middle circles depict the GC-skew and GC content, respectively. The outermost circle indicates the arrangements of genes: inner genes from the reverse strand, and outer genes from the forward strand, with PCGs in bluish violet, rRNAs in light green, and tRNAs in light purple. Different colors are used to show different functional categories, as shown in the upper right of the picture.

The whole mitochondrial genome of *O. phaseoli* has a total length of 15,540 bp (GenBank: OR593754; NCBI Reference Sequence: NC084197), including 37 genes and two putative control regions (CR1 and CR2) (Figure 2). Six genes (*nad5*, *nad4*, *nad4L*, *trnH*, *trnV* and *trnP*) are encoded in the L-strand, while the others were encoded in the H-strand (Table S1). There are 18 intergenic spaces and six pairs of genes overlap in the entire mitogenome (Table S1). The nucleotide composition of this mitogenome was 78.8% A + T content. The total length of the 13 PCGs, ranging from 160 bp (*atp8*) to 1695 bp (*nad5*), was 10,953bp, in which all PCGs used ATN start codons, but most of them used TAA or TAG as a stop codon while four genes (*atp8*, *nad1*, *nad2* and *nad4*) have an incomplete ‘T’. All 22 tRNAs were found in this mitogenome, with a total length of 1,435bp ranging from 56 bp (*trnS1*) to 80 bp (*trnV*) in size (Table S1). Most tRNAs have a typical clover-leaf secondary structure with one exception, *trnS1*, lacking the DHU arm (Figure S2). Two rRNA genes have a full length of 775 bp in *rrnS* which is located between *trnF* and *cox2*, and of 1,088bp in *rrnL* that is located between *trnN* and *trnS2*. The coverage depth of *O. phaseoli* is shown in Figure S1.

**Figure 3.**
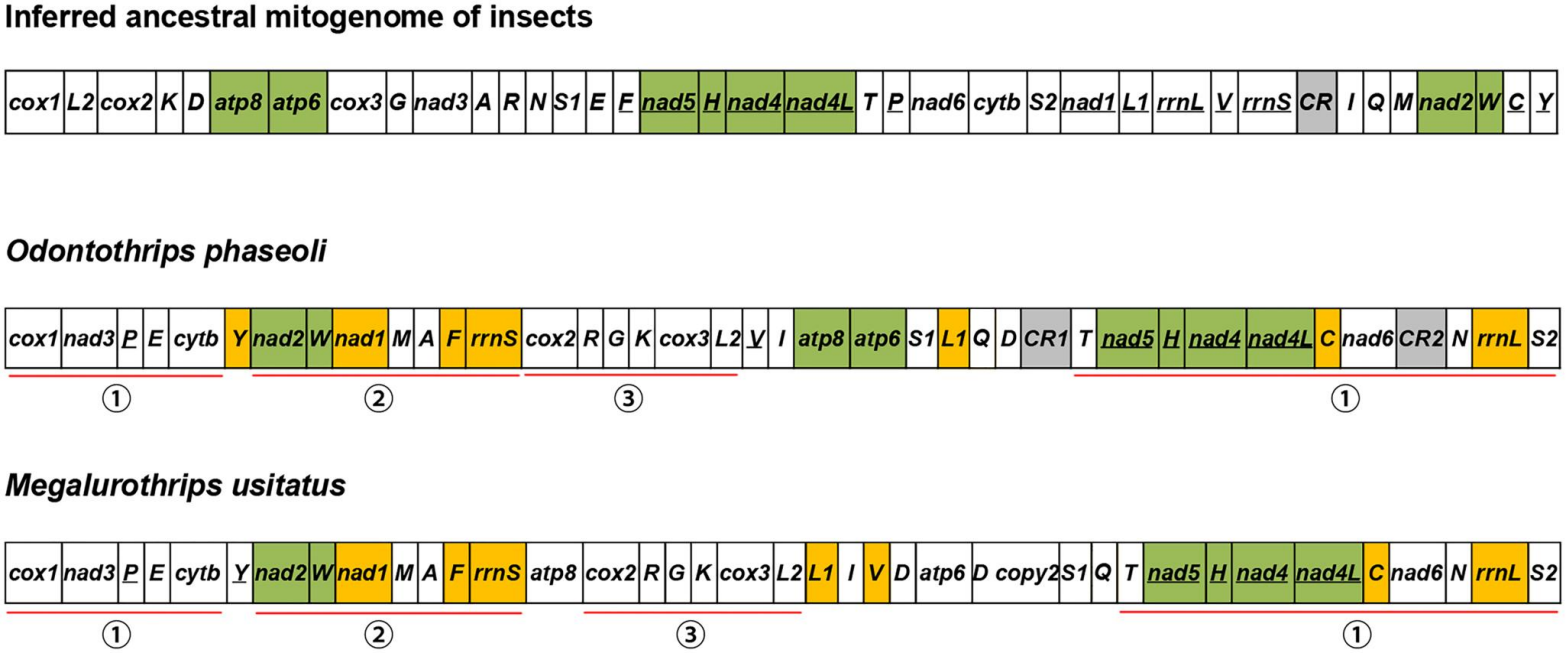
Mitochondrial gene arrangements in the ancestor insect, *O. phaseoli*, and *M. usitatus*. Conserved gene blocks are highlighted in green, and the inverted genes in orange.

When comparing the mitochondrial gene order of *O. phaseoli* to the inferred insect ancestral arrangement it displayed significant rearrangement differences (Clary and Wolstenholme 1985) (Figure 3). But three gene blocks (*atp8-atp6*, *nad5-trnH-nad4-nad4L* and *nad2-trnW*) are conserved in *O. phaseoli* (Figure 3). A comparison between the mitochondrial gene arrangements of *O. phaseoli* and its related species, *Megalurothrips usitatus*, shows that they both share three conserved gene blocks (Figure 3 - ①, ②, ③).

**Figure 4.**
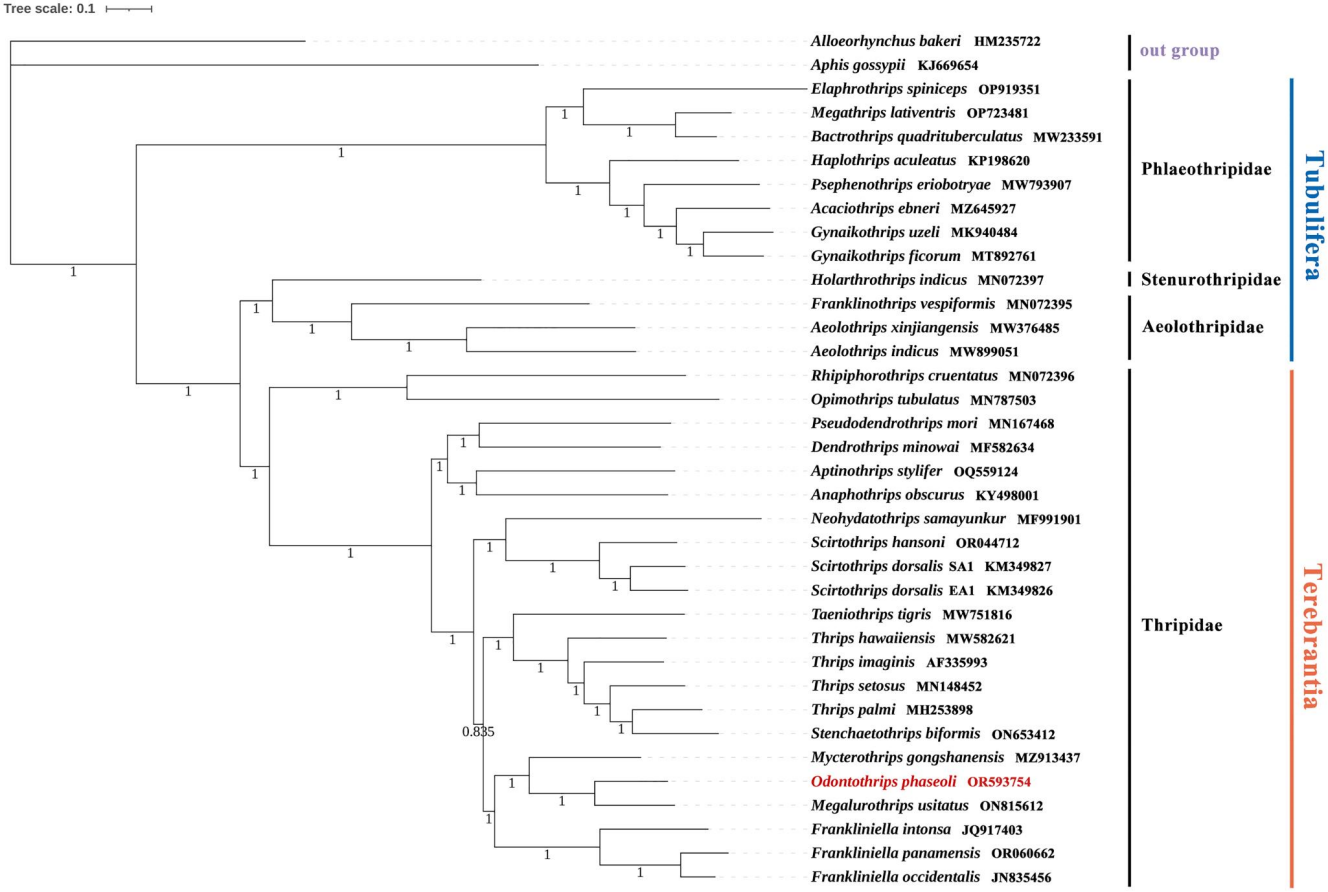
BI phylogenetic tree inferred from the whole mitogenome. The tree was constructed based on concatenated nucleotide sequences of the whole mitogenome dataset (13PCG + 22tRNA + 2rRNA) of 36 species in 6 families. The numbers under the internodes represent Bayesian inference (BI) posterior probabilities (PP). The scale bar refers to 0.1 nucleotide substitutions per character. The used sequences and their references were listed in (Table S3): *Alloeorhynchus bakeri* (HM235722; Li et al. 2012), *Aphis gossypii* (KJ669654; Zhang et al. 2016), *Acaciothrips ebneri* (MZ645927; not available), *Bactrothrips quadrituberculatus* (MW233591; not available), *Elaphrothrips spiniceps* (OP919351; not available), *Megathrips lativentris* (OP723481; not available), *Gynaikothrips ficorum* (MT892761; Dang et al. 2021), *Gynaikothrips uzeli* (MK940484; Tyagi et al. 2020), *Haplothrips aculeatus* (KP198620; not available), *Psephenothrips eriobotryae* (MW793907; Dang et al. 2024), *Holarthrothrips indicus* (MN072397; Tyagi et al. 2020), *Aeolothrips indicus* (MW899051; Pakrashi et al. 2021), *Aeolothrips xinjiangensis* (MW376485; Liu et al. 2022), *Franklinothrips vespiformis* (MN072395; Tyagi et al. 2020), *Anaphothrips obscurus* (KY498001; Liu et al. 2017), *Frankliniella panamensis* (OR060662; not available), *Dendrothrips minowai* (MF582634; Chen et al. 2017), *Frankliniella intonsa* (JQ917403; Yan et al. 2014), *Frankliniella occidentalis* (JN835456; Yan et al. 2012), *Scirtothrips hansoni* (OR044712; not available), *Neohydatothrips samayunkur* (MF991901; Kumar et al. 2019), *Mycterothrips gongshanensis* (MZ913437; not available), *Rhipiphorothrips cruentatus* (MN072396;Tyagi et al. 2020), *Scirtothrips dorsalis* EA1 (KM349826; Dickey et al. 2015), *Scirtothrips dorsalis* SA1 (KM349827, KM349828; Dickey et al. 2015), *Odontothrips phaseoli* (OR593754; this study), *Stenchaetothrips biformis* (ON653412; Hu et al. 2023), *Taeniothrips tigris* (MW751816; Pakrashi et al. 2021), *Thrips imagines* (AF335993; Shao and Barker 2003), *Thrips hawaiiensis* (MW582621; Wang et al. 2021), *Thrips palmi* (MH253898; Chakraborty et al. 2018), *Thrips setosus* (MN148452; not available), *Opimothrips tubulatus* (MN787503; not available), *Megalurothrips usitatus* (ON815612; Xing-Ming et al. 2023), *Aptinothrips stylifer* (OQ559124; Li et al. 2024), *Pseudodendrothrips mori* (MN167468; not available).

The phylogeny confirms the monophyly of two suborders, Tubulifera and Terebrantia. Moreover, *O. phaseoli* was clustered to *Megalurothrips usitatus* with a high support value (PP = 1), which is consistent with previous phylogenetic studies (Figure 4) (Mound and Palmer 1981). Especially, the results of DNA barcoding studies supported that these two species were also close related (Rebijith et al. 2014). The two genera that belong to the *Megalurothrips* genus-group share the morphological characteristics: antennae 8-segmented, ocellar setae pair I present, median metanotal setae at anterior margin, metanotal spinula absent, tergite VIII with posteromarginal comb usually interrupted, and sternal discal setae absent, which might be a synapomorphy for this group (Hakimara and Minaei 2019).

## Discussion and conclusion

In this study, the complete mitochondrial genome of *Odontothrips phaseoli* was characterized, in which 37 genes and two putative CRs are recognized in a total length of 15,540bp. Compared to the primitive ancestral mitogenome of arthropods, *O. phaseoli* displayed significant gene rearrangement differences. In addition, there is a noticeable similarity in the occurrence of gene transpositions between *O. phaseoli* and *M. usitatus*. The phylogenetic tree showed that *O. phaseoli* was robustly clustered with *M. usitatus*, affirming its affiliation within the *Megalurothrips* genus-group. Furthermore, this alignment might underscore the close link between gene arrangements and the phylogeny relationships.

## Supplementary Material

Table S1.docx

Figure S2.docx

Table S3.docx

Table S2.docx

Figure S1.docx

## Data Availability

The data that support the findings of this study are openly available in GenBank at https://www.ncbi.nlm.nih.gov/genbank/ under the accession no. OR593754. The associated BioProject, Bio-Sample numbers, and SRA are PRJNA1021028, SAMN37542142, and SRR26222581, respectively.
