# Supplementary material for: Characterizing the complete mitogenome of Odontothrips phaseoli (Thysanoptera: Thripidae) and its mitochondrial phylogeny
Source: Mitochondrial DNA B Resour. 2024 Jul 31;9(8):965–70. doi: 10.1080/23802359.2024.2386418 (PMC11293258; doi:10.1080/23802359.2024.2386418)
Supplement: Table S1.docx [file TMDN_A_2386418_SM8485.docx]

**Table S1** Organization of the mitochondrial genome of *Odontothrips phaseoli*. Intergenic nucleotides described as intergenic (+) or overlapping nucleotides (-). (H) in strand represent as heavy and (L) as light. CR represents the control region.

| Gene | Position | | Size | Intergenic nucleotides | Codon | | Strand | AT content |
| --- | --- | --- | --- | --- | --- | --- | --- | --- |
|  | From | To |  |  | Start | Stop |  |  |
| *cox1* | 1 | 1542 | 1542 |  | ATG | TAA | H | 72.9 |
| *nad3* | 1546 | 1899 | 354 | 3 | ATT | TAA | H | 79.1 |
| *trnP* | 1920 | 1981 | 62 | 20 |  |  | L | 80.6 |
| *trnE* | 1985 | 2048 | 64 | 3 |  |  | H | 87.5 |
| *cytb* | 2066 | 3166 | 1101 | 17 | ATA | TAA | H | 74.4 |
| *trnY* | 3200 | 3265 | 66 | 33 |  |  | H | 83.3 |
| *nad2* | 3299 | 4274 | 976 | 33 | ATA | T | H | 82.8 |
| *trnW* | 4275 | 4339 | 65 |  |  |  | H | 87.7 |
| *nad1* | 4340 | 5261 | 922 |  | ATA | T | H | 77.4 |
| *trnM* | 5263 | 5324 | 62 | 1 |  |  | H | 74.2 |
| *trnA* | 5325 | 5389 | 65 |  |  |  | H | 89.2 |
| *trnF* | 5415 | 5478 | 64 | 25 |  |  | H | 81.2 |
| *rrnS* | 5479 | 6253 | 775 |  |  |  | H | 80.8 |
| *cox2* | 6254 | 6904 | 651 |  | ATG | TAA | H | 75.3 |
| *trnR* | 6909 | 6976 | 68 | 4 |  |  | H | 73.5 |
| *trnG* | 6976 | 7037 | 62 | -1 |  |  | H | 74.2 |
| *trnK* | 7038 | 7099 | 62 |  |  |  | H | 82.3 |
| *cox3* | 7110 | 7892 | 783 | 10 | ATT | TAA | H | 73.5 |
| *trnL2* | 7896 | 7960 | 65 | 3 |  |  | H | 84.6 |
| *trnV* | 7940 | 8019 | 80 | -21 |  |  | L | 80 |
| *trnI* | 8283 | 8351 | 69 | 263 |  |  | H | 69.6 |
| *atp8* | 8443 | 8602 | 160 | 91 | ATT | T | H | 85 |
| *atp6* | 8575 | 9264 | 690 | -28 | ATA | TAA | H | 77.2 |
| *trnS1* | 9315 | 9370 | 56 | 50 |  |  | H | 80.4 |
| *trnL1* | 9372 | 9436 | 65 | 1 |  |  | H | 75.4 |
| *trnQ* | 9492 | 9559 | 68 | 55 |  |  | H | 88.2 |
| *trnD* | 9580 | 9646 | 67 | 20 |  |  | H | 80.6 |
| *CR1* | 9647 | 10086 | 440 |  |  |  | H | 82 |
| *trnT* | 10087 | 10152 | 66 |  |  |  | H | 84.8 |
| *nad5* | 10144 | 11838 | 1695 | -9 | ATA | TAA | L | 79.2 |
| *trnH* | 11839 | 11903 | 65 |  |  |  | L | 84.6 |
| *nad4* | 11904 | 13221 | 1318 |  | ATT | T | L | 77.9 |
| *nad4L* | 13215 | 13511 | 297 | -7 | ATG | TAA | L | 83.2 |
| *trnC* | 13488 | 13550 | 63 | -24 |  |  | H | 84.1 |
| *nad6* | 13577 | 14044 | 468 | 26 | ATT | TAG | H | 80.5 |
| *CR2* | 14045 | 14318 | 274 |  |  |  | H | 91.6 |
| *trnN* | 14319 | 14384 | 66 |  |  |  | H | 80.8 |
| *rrnL* | 14385 | 15472 | 1088 |  |  |  | H | 81 |
| *trnS2* | 15473 | 15537 | 65 |  |  |  | H | 86.2 |
