# Supplementary figures and images for: Characterizing the complete mitogenome of Odontothrips phaseoli (Thysanoptera: Thripidae) and its mitochondrial phylogeny
Source: Mitochondrial DNA B Resour. 2024 Jul 31;9(8):965–70. doi: 10.1080/23802359.2024.2386418 (PMC11293258; doi:10.1080/23802359.2024.2386418)

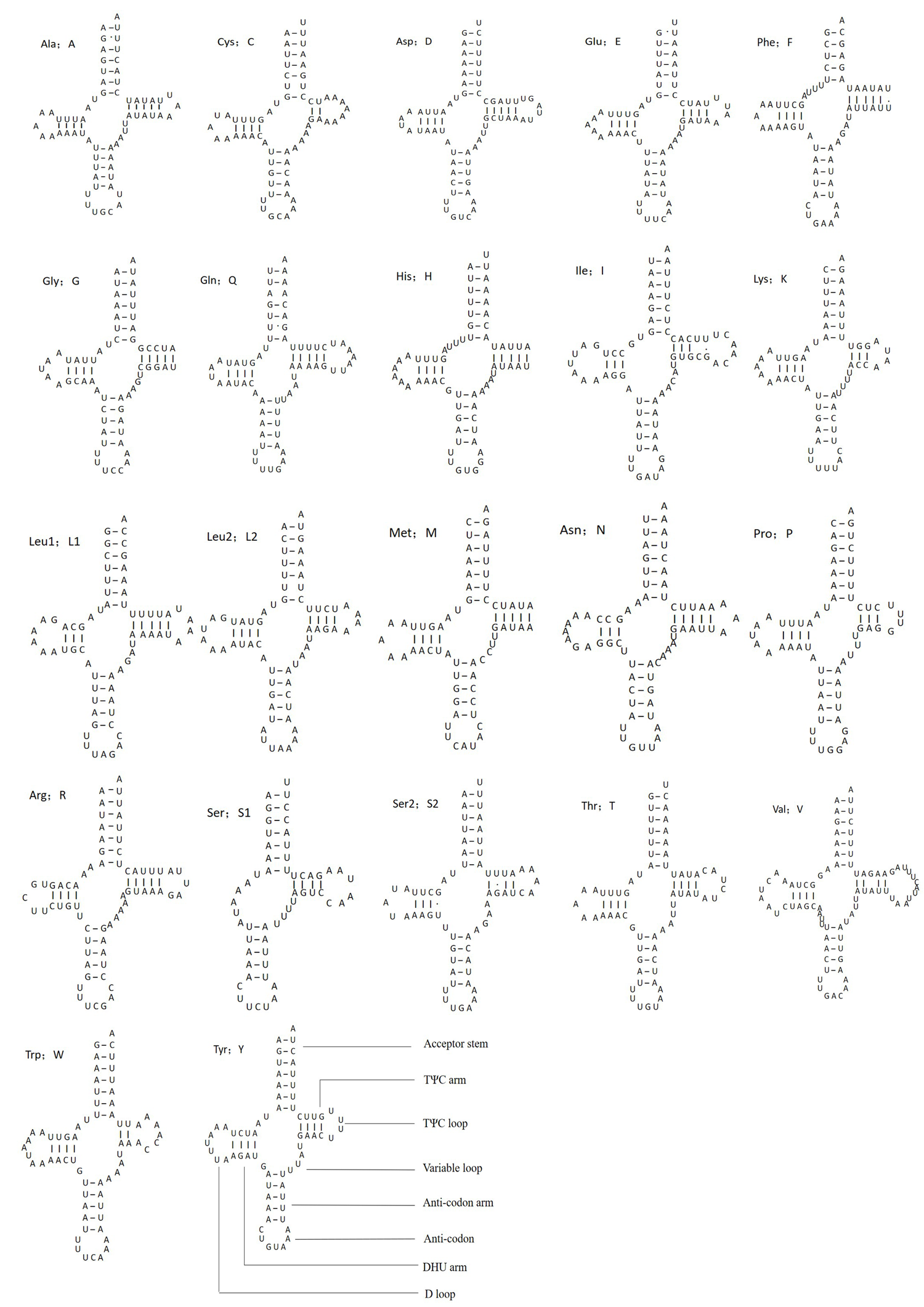


Figure S2. Putative cloverleaf secondary structures of the 22 tRNAs of *Odontothrips phaseoli*.

Supplement: Figure S2.docx [file TMDN_A_2386418_SM8484.docx]

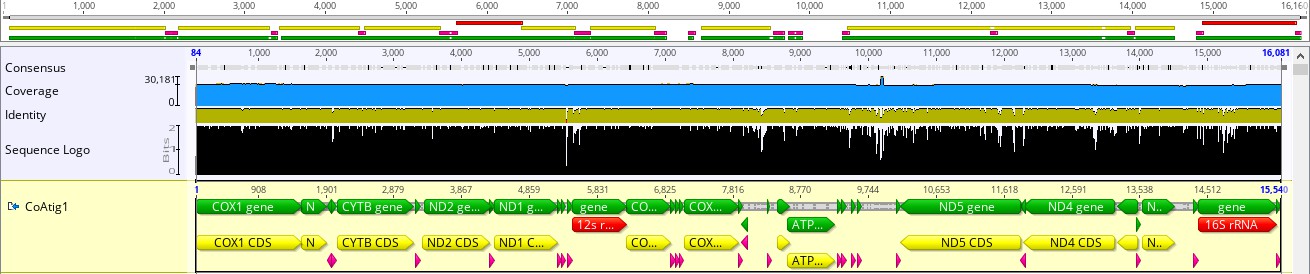


Figure S1. Read coverage plot for studies using high throughput DNA sequencing.

Supplement: Figure S1.docx [file TMDN_A_2386418_SM8481.docx]
