# Supplementary material for: Characterizing the complete mitogenome of Odontothrips phaseoli (Thysanoptera: Thripidae) and its mitochondrial phylogeny
Source: Mitochondrial DNA B Resour. 2024 Jul 31;9(8):965–70. doi: 10.1080/23802359.2024.2386418 (PMC11293258; doi:10.1080/23802359.2024.2386418)
Supplement: Table S3.docx [file TMDN_A_2386418_SM8483.docx]

**Table S3.** List of species included in the phylogenetic analysis.

| **Order** | **Family** | **Species** | **GenBank accession number** | **References** | | | |
| --- | --- | --- | --- | --- | --- | --- | --- |
| **Outgroup** |  |  |  |  |  |  |  |
| Hemiptera | Nabidae | *Alloeorhynchus bakeri* | HM235722 | Li et al.,2012 | | | |
|  | Aphididae | *Aphis gossypii* | KJ669654 | Zhang et al.,2016 | | | |
| **Ingroup** |  |  |  |  |  |  |  |
| Thysanoptera | Phlaeothripidae | *Acaciothrips ebneri* | MZ645927 | Not available | | | |
|  |  | *Bactrothrips quadrituberculatus* | MW233591 | Not available | | | |
|  |  | *Elaphrothrips spiniceps* | OP919351 | Not available | | | |
|  |  | *Megathrips lativentris* | OP723481 | Not available | | | |
|  |  | *Gynaikothrips ficorum* | MT892761 | Dang et al.,2021 | | | |
|  |  | *Gynaikothrips uzeli* | MK940484 | Tyagi et al.,2020 | | | |
|  |  | *Haplothrips aculeatus* | KP198620 | Not available | | | |
|  |  | *Psephenothrips eriobotryae* | MW793907 | Dang et al.,2024 | | | |
|  | Stenurothripidae | *Holarthrothrips indicus* | MN072397 | Tyagi et al.,2020 | | | |
|  | Aeolothripidae | *Aeolothrips indicus* | MW899051 | Pakrashi et al.,2021 | | | |
|  |  | *Aeolothrips xinjiangensis* | MW376485 | Liu et al.,2022 | | | |
|  |  | *Franklinothrips vespiformis* | MN072395 | Tyagi et al.,2020 | | | |
|  | Thripidae | *Anaphothrips obscurus* | KY498001 | Liu et al.,2017 | | | |
|  |  | *Frankliniella panamensis* | OR060662 | Not available | | | |
|  |  | *Dendrothrips minowai* | MF582634 | Chen et al.,2017 | | | |
|  |  | *Frankliniella intonsa* | JQ917403 | Yan et al.,2014 | | | |
|  |  | *Frankliniella occidentalis* | JN835456 | Yan et al.,2012 | | | |
|  |  | *Scirtothrips hansoni* | OR044712 | Not available | | | |
|  |  | *Neohydatothrips samayunkur* | MF991901 | Kumar et al.,2019 | | | |
|  |  | *Mycterothrips gongshanensis* | MZ913437 | Not available | | | |
|  |  | *Rhipiphorothrips cruentatus* | MN072396 | Tyagi et al.,2020 | | | |
|  |  | *Scirtothrips dorsalis* EA1 | KM349826 | Dickey et al.,2015 | | | |
|  |  | *Scirtothrips dorsalis* SA1 | KM349827  KM349828 | Dickey et al.,2015 | | | |
|  |  | ***Odontothrips phaseoli*** | **OR593754** | **This study** | | | |
|  |  | *Stenchaetothrips biformis* | ON653412 | Hu et al.,2023 | | | |
|  |  | *Taeniothrips tigris* | MW751816 | Pakrashi et al.,2021 | | | |
|  |  | *Thrips imaginis* | AF335993 | Shao et al.,2003 | | | |
|  |  | *Thrips hawaiiensis* | MW582621 | Wang et al.,2021 | | | |
|  |  | *Thrips palmi* | MH253898 | Chakraborty et al.,2018 | | | |
|  |  | *Thrips setosus* | MN148452 | Not available | | | |
|  |  | *Opimothrips tubulatus* | MN787503 | Not available | | | |
|  |  | *Megalurothrips usitatus* | ON815612 | Lin et al.,2023 | | | |
|  |  | *Aptinothrips stylifer* | OQ559124 | Li et al.,2024 | | | |
|  |  | *Pseudodendrothrips mori* | MN167468 | Not available | | | |
