# Supplementary material for: Characterizing the complete mitogenome of Odontothrips phaseoli (Thysanoptera: Thripidae) and its mitochondrial phylogeny
Source: Mitochondrial DNA B Resour. 2024 Jul 31;9(8):965–70. doi: 10.1080/23802359.2024.2386418 (PMC11293258; doi:10.1080/23802359.2024.2386418)
Supplement: Table S2.docx [file TMDN_A_2386418_SM8482.docx]

**Table S2.** The codon usage of mitochondrial genome of *Odontothrips phaseoli*

| Codon | Count | RSCU | Codon | Count | RSCU | Codon | Count | RSCU | Codon | Count | RSCU |
| --- | --- | --- | --- | --- | --- | --- | --- | --- | --- | --- | --- |
| UUU(F) | 427 | 1.75 | UCU(S) | 130 | 2.74 | UAU(Y) | 121 | 1.67 | UGU(C) | 38 | 1.81 |
| UUC(F) | 60 | 0.25 | UCC(S) | 17 | 0.36 | UAC(Y) | 24 | 0.33 | UGC(C) | 4 | 0.19 |
| UUA(L) | 333 | 3.94 | UCA(S) | 117 | 2.47 | UAA(*) | 8 | 1.78 | UGA(W) | 68 | 1.79 |
| UUG(L) | 35 | 0.41 | UCG(S) | 7 | 0.15 | UAG(*) | 1 | 0.22 | UGG(W) | 8 | 0.21 |
| CUU(L) | 85 | 1.01 | CCU(P) | 41 | 1.5 | CAU(H) | 47 | 1.59 | CGU(R) | 13 | 1.06 |
| CUC(L) | 6 | 0.07 | CCC(P) | 11 | 0.4 | CAC(H) | 12 | 0.41 | CGC(R) | 0 | 0 |
| CUA(L) | 46 | 0.54 | CCA(P) | 55 | 2.02 | CAA(Q) | 46 | 1.77 | CGA(R) | 35 | 2.86 |
| CUG(L) | 2 | 0.02 | CCG(P) | 2 | 0.07 | CAG(Q) | 6 | 0.23 | CGG(R) | 1 | 0.08 |
| AUU(I) | 337 | 1.76 | ACU(T) | 59 | 1.49 | AAU(N) | 123 | 1.47 | AGU(S) | 22 | 0.46 |
| AUC(I) | 45 | 0.24 | ACC(T) | 13 | 0.33 | AAC(N) | 44 | 0.53 | AGC(S) | 0 | 0 |
| AUA(M) | 247 | 1.85 | ACA(T) | 85 | 2.15 | AAA(K) | 150 | 1.79 | AGA(S) | 79 | 1.67 |
| AUG(M) | 20 | 0.15 | ACG(T) | 1 | 0.03 | AAG(K) | 18 | 0.21 | AGG(S) | 7 | 0.15 |
| GUU(V) | 97 | 2.28 | GCU(A) | 52 | 2 | GAU(D) | 49 | 1.56 | GGU(G) | 59 | 1.35 |
| GUC(V) | 4 | 0.09 | GCC(A) | 6 | 0.23 | GAC(D) | 14 | 0.44 | GGC(G) | 1 | 0.02 |
| GUA(V) | 67 | 1.58 | GCA(A) | 46 | 1.77 | GAA(E) | 77 | 1.86 | GGA(G) | 104 | 2.38 |
| GUG(V) | 2 | 0.05 | GCG(A) | 0 | 0 | GAG(E) | 6 | 0.14 | GGG(G) | 11 | 0.25 |
